# Brain heart crosstalk after ischemic stroke toward a unified framework for pathophysiology and precision medicine

**DOI:** 10.3389/fnmol.2026.1889923

**Published:** 2026-07-24

**Authors:** Pengpeng Li, Yangyang Gao, Wei Liu

**Affiliations:** Xi’an Aerospace Hospital of Northwest University, Xi’an, Shanxi, China

**Keywords:** brain-heart axis, cardiovascular dysfunction, ischemic stroke, neuroinflammation, stroke

## Abstract

**Background:**

Acute ischemic stroke and cardiac dysfunction engage in a complex bidirectional interplay that critically determines patient outcomes. Despite growing recognition of the brain-heart axis, a comprehensive mechanistic framework integrating traditional and emerging pathways remains lacking. Moreover, existing reviews have predominantly focused on descriptive mechanisms without systematically evaluating the strength of evidence, addressing knowledge gaps, or proposing testable hypotheses for precision medicine.

**Recent advances:**

This review systematically delineates the pathophysiology, clinical spectrum, and management of brain-heart crosstalk following acute ischemic stroke. We first establish the core mechanisms: autonomic dysregulation with catecholamine surge, hypothalamic pituitary adrenal axis activation, neuroinflammation and systemic immune suppression, mechanical coupling via mechanosensitive Piezo1 and Piezo2 channels, and the gut brain-heart axis mediated by trimethylamine N oxide. Critically, we identify where evidence is robust versus preliminary, highlighting inconsistencies such as the divergent roles of Piezo1 versus Piezo2 in cardiac pathology and the unresolved causal relationship between trimethylamine N oxide and post stroke outcomes. We then examine the clinical consequences including Takotsubo syndrome, atrial fibrillation, QT prolongation, left ventricular dysfunction, and secondary brain injury, and critically appraise current management strategies such as hemodynamic control, beta blockers, anti-inflammatory agents, and neuromodulation.

**Key challenges and future directions:**

Major knowledge gaps persist regarding the temporal dynamics of autonomic dysregulation, the narrow therapeutic window for beta blockade in acute stroke, the lack of stroke specific anti-inflammatory trials, and the underexplored potential of gut microbiota modulation as a therapeutic target. We propose a testable precision medicine paradigm integrating multimodal biomarkers to identify high risk individuals and guide targeted interventions.

**Conclusion:**

The brain-heart axis is a pivotal determinant of post stroke prognosis. However, current evidence remains predominantly observational or derived from non-stroke populations. Moving beyond descriptive integration toward mechanism based, hypothesis driven, and personalized strategies will be essential to improve long term outcomes in this vulnerable population.

## 1 Introduction

The most recent Global Burden of Disease Study (GBD) 2021 reports that in 2021, there were 11.9 million new incident strokes and 93.8 million prevalent stroke survivors globally, with 7.3 million deaths attributable to stroke, ranking it the third leading cause of death worldwide and the fourth leading cause of disability-adjusted life years (GBD 2021 Stroke Risk Factor Collaborators, 2024). A more nuanced assessment emerges when applying the disability-adjusted life years (DALYs) metric, wherein stroke ascends to the third greatest contributor to global health loss (Feigin et al., 2025). This sobering reality has intensified research into determinants of post-stroke outcomes, thrusting brain-heart interactions into the spotlight as a pivotal frontier in clinical investigation.

Crucially, not all ischemic strokes confer equivalent cardiac risk. The brain-heart axis has been most extensively characterized in stroke phenotypes that disrupt central autonomic networks, particularly right insular cortex lesions and large hemispheric infarcts. The insular cortex serves as a primary hub for viscerosensory integration and sympathetic cardiovascular regulation, with the right hemisphere exhibiting dominance for this function. Right insular infarction is associated with substantially higher rates of cardiac troponin elevation, increased plasma catecholamines, and malignant arrhythmias compared with strokes in other territories (Li H. et al., 2026). Large hemispheric infarcts involving the anterior cingulate, amygdala, or hypothalamic nuclei produce similarly profound dysautonomia, whereas lacunar and small subcortical strokes that spare these structures are not associated with significant cardiac complications (Celaj and Prabhakaran, 2019). Brainstem strokes affecting the nucleus tractus solitarius or ventrolateral medulla represent another high-risk phenotype, characterized by baroreflex failure and extreme blood pressure lability. This topographic specificity mandates a lesion-informed rather than unitary approach to post-stroke cardiac risk stratification.

Stroke survivors, particularly those with high-risk phenotypes such as right insular or large hemispheric infarction, face a substantial risk of developing a spectrum of cardiovascular complications (Chen et al., 2017). These manifestations range from life-threatening conditions, such as acute myocardial injury and acute coronary syndrome, to distinctive pathological entities like left ventricular dysfunction, particularly the characteristic apical ballooning presentation of Takotsubo cardiomyopathy (Scheitz et al., 2022). Of particular concern is the approximately 15% of patients who may experience sudden malignant arrhythmias, with some cases progressing to sudden cardiac death (Hoad et al., 2025). Beyond these overt clinical presentations, a considerable proportion of patients exhibit only subclinical abnormalities, including elevated cardiac injury biomarkers or subtle electrocardiographic modifications (Johansen et al., 2024). Critically, these cardiovascular complications demonstrate a strong association with more severe neurological deficits and elevated mortality rates during the acute stroke phase (Mikulenka et al., 2024).

Accumulating evidence supports the existence of a sophisticated bidirectional communication network between the brain and the heart, known as the “brain-heart axis” (Valenza et al., 2025). This anatomical and functional pathway underpins the continuous interaction through which the nervous and cardiovascular systems modulate human physiology, cognition, and emotion. The brain-heart axis is increasingly recognized as a critical physiological system that is frequently disrupted in disease states (Simats et al., 2024a). Even in the absence of pre-existing cardiac conditions, acute brain injury, particularly when involving autonomic regulatory centers such as the insular cortex or brainstem cardiovascular nuclei, can precipitate cardiac dysfunction, thereby elevating mortality risk and potentially leading to long-term cardiac sequelae such as heart failure (Ziaka and Exadaktylos, 2023). Conversely, impaired cardiac function can exacerbate cerebral injury, establishing a self-perpetuating cycle of deterioration. In patients with cardiovascular dysfunction, research has predominantly focused on hemodynamic impairment resulting from arrhythmias, regional wall motion abnormalities, or left ventricular hypokinesia that compromise cerebral perfusion pressure. However, understanding of the potential secondary and delayed cerebral complications remains limited. Notably, a growing body of evidence has revealed that cognitive impairment frequently manifests in patients diagnosed with heart failure (van Nieuwkerk et al., 2023).

Despite these advances, substantial challenges persist in clinical management, and the existing literature suffers from several critical limitations. First, most mechanistic studies derive from animal models with questionable translatability to human stroke, particularly regarding the timing and magnitude of catecholamine surges; moreover, these models typically employ middle cerebral artery occlusion that may not fully recapitulate the topographic specificity observed in human stroke patients. Second, clinical evidence is largely observational, with few randomized controlled trials specifically targeting brain-heart crosstalk. Third, the relative contribution of each proposed mechanism including autonomic, inflammatory, mechanical, and gut derived pathways remains undefined in human patients, and their interactions are almost certainly synergistic rather than additive. Fourth, diagnostic limitations are particularly evident, as post stroke cardiac complications frequently evade detection owing to atypical presentation, a predicament especially prevalent among elderly and diabetic populations (Wang et al., 2024). Therapeutic strategies remain equally constrained by the absence of specific guidelines targeting brain heart crosstalk, compelling clinicians to adapt protocols designed for isolated cardiac or neurological conditions, often with suboptimal outcomes (Buckley et al., 2022). The stroke heart syndrome has been conclusively linked to long term major adverse cardiovascular events in ischemic stroke survivors (Choi et al., 2024).

More fundamentally, critical knowledge gaps persist in our mechanistic understanding. The molecular pathways underlying chronic brain-heart comorbidity remain elusive, while the intricate interplay between autonomic regulation and neuroimmune responses demands further elucidation (Moura et al., 2024). Specifically, it remains unknown whether autonomic dysfunction is the primary driver or merely an epiphenomenon of severe stroke, whether anti-inflammatory interventions that are effective in chronic coronary disease can be safely applied in the acute post stroke setting, and whether trimethylamine N oxide elevation is causal or merely a marker of gut dysbiosis following stroke. The mediating role of the gut microbiota within this network similarly awaits definitive confirmation (Zhang et al., 2024). Addressing these multifaceted questions necessitates transcending traditional disciplinary boundaries through the integration of perspectives from neuroscience, cardiology, and immunology. This review aims to systematically synthesize current knowledge, critically evaluate the strength of available evidence, and identify unresolved controversies. We also propose a testable framework for future hypothesis-driven research. This framework addresses the pathophysiology, clinical implications, and therapeutic strategies of brain-heart interactions.

## 2 Pathophysiological mechanisms of brain-heart crosstalk

Brain-heart crosstalk following ischemic stroke is mediated by a complex network of interconnected mechanisms that operate across distinct temporal phases. These pathways can be broadly categorized according to their temporal profiles. Acute mechanisms, unfolding within hours to days, encompass autonomic dysregulation with catecholamine surge and baroreceptor dysfunction, which are primarily responsible for early cardiac complications. Subacute mechanisms, evolving over days to weeks, include neuroinflammation, HPA axis activation, and systemic immune responses. Chronic mechanisms, manifesting over weeks to months, particularly involve the gut-brain-heart axis through TMAO-mediated pathways and persistent low-grade inflammation, which contribute to long-term cardiovascular risk and atherosclerosis progression. Understanding these temporal distinctions is essential for identifying appropriate therapeutic windows and guiding clinical management.

**FIGURE 1:**
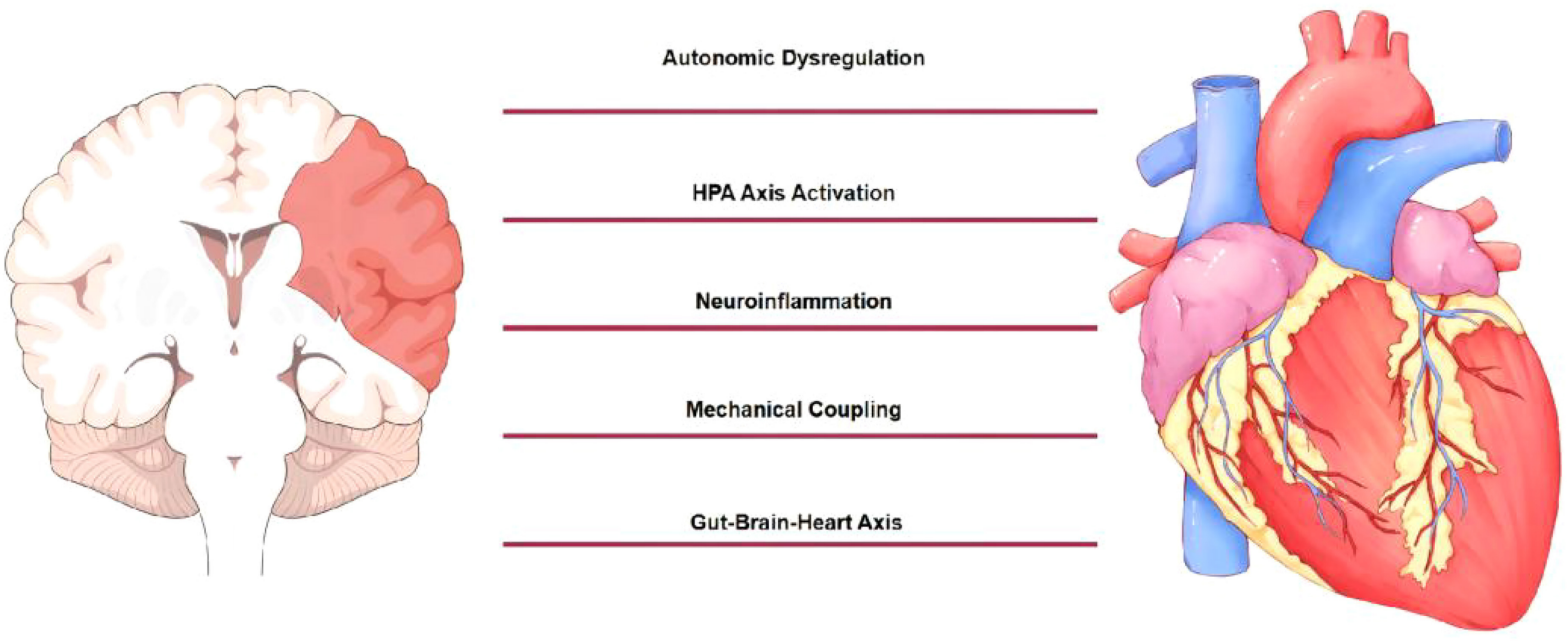
Schematic overview of the brain-heart axis following ischemic stroke. This diagram illustrates the five core mechanisms linking the brain and heart after ischemic stroke. Autonomic dysregulation drives catecholamine surge and sympathetic overactivation, directly injuring cardiomyocytes. HPA axis activation releases cortisol and pro-inflammatory cytokines, amplifying systemic inflammation. Neuroinflammation is mediated by microglial activation and peripheral immune infiltration. Mechanical coupling operates through Piezo1/2 channels and baroreceptor dysfunction. Gut-brain-heart axis involves microbiota-derived metabolites such as TMAO, promoting thrombosis and inflammation. These interconnected pathways converge to induce cardiac complications including Takotsubo syndrome, arrhythmias, and left ventricular dysfunction.

Brain heart crosstalk following ischemic stroke is mediated by a complex network of interconnected mechanisms. However, it is important to recognize that the majority of evidence derives from animal models, primarily rodent middle cerebral artery occlusion, and observational human studies. Causal relationships in human patients remain largely inferred rather than proven, and the temporal hierarchy of these mechanisms, specifically which pathway activates first and which dominates at different post stroke stages, has not been systematically established. A schematic overview of these pathways is presented in Figure 1.

### 2.1 Autonomic nervous system dysregulation

The insular cortex, anterior cingulate cortex, and amygdala orchestrate higher-order autonomic control. Evidence derived from human neuroimaging and clinical observational studies indicates that lesions in these regions can provoke systematic dysautonomia of varying severity (Kang et al., 2024). Complementing these clinical observations, experimental studies have rigorously interrogated the causal relationship between specific brain lesions and cardiovascular outcomes. Using targeted pharmacological and optogenetic manipulations in rodent models, Colombari et al. (2025) demonstrated that disruption of hypothalamic and brainstem circuits involved in cardiovascular homeostasis produces distinct patterns of cardiac autonomic dysregulation, with these findings originating from animal models. These experimental approaches are particularly valuable for dissecting the relative contributions of sympathetic versus parasympathetic pathways to post-stroke cardiac pathology, a distinction difficult to achieve in human observational studies and thus requiring integration of evidence from both human studies and animal models (Inamasu et al., 2013).

Studies in animal models show that cerebral ischemia leads to proportional increases in plasma catecholamines. This response directly correlates with the development of cardiomyopathy and cardiac injury (Hachinski et al., 1986). However, important caveats exist: most animal studies measure catecholamines within hours of stroke onset, whereas human data suggest substantial inter individual variability that is poorly explained by infarct location alone. Furthermore, the threshold of catecholamine elevation required to produce clinically significant cardiac injury remains undefined. Once released, catecholamines such as epinephrine and norepinephrine act through β-adrenergic receptor signaling. They induce myocardial calcium overload, mitochondrial dysfunction, and contraction band necrosis, pathological changes that have been characterized primarily in animal models and ex vivo studies, with limited pathological confirmation in human tissue. These pathological changes may ultimately manifest as Takotsubo syndrome or lethal arrhythmias, including QT prolongation and ventricular fibrillation, clinical manifestations that are well-documented in human observational studies (Singh et al., 2022).

Takotsubo syndrome, characterized by transient left ventricular systolic dysfunction, often presents a diagnostic challenge indistinguishable from acute myocardial infarction in human clinical practice (Omerovic et al., 2022). Although historically considered a benign, self-limiting condition, contemporary evidence confirms persistent subtle cardiac dysfunction in these patients. Despite restored left ventricular ejection fraction, many experience restrictive symptoms, as demonstrated in human cohort studies (Templin et al., 2015). Moreover, these patients carry substantial morbidity and mortality burdens, with subsequent major adverse cardiovascular event rates approaching those observed in acute coronary syndrome populations according to human registry data (Redfors et al., 2021). It remains to be determined whether genetic susceptibility, pre-existing cardiac reserve, or concurrent inflammatory state modulate individual risk, as only a minority of stroke patients with comparable insular lesions develop Takotsubo syndrome.

### 2.2 Cardiocerebral mechanical coupling and hemodynamics

The cardiac mechanical impulse serves not only as a pump maintaining blood circulation but also generates periodic pulse waves that function as crucial trans-organ communication signals, a concept supported by both human physiological studies and animal models (Ince et al., 2020). This pathway relies fundamentally on mechanosensitive ion channels Piezo1 and Piezo2, which have been characterized primarily through animal models and *ex vivo* electrophysiological studies (Coste et al., 2010). Under physiological conditions, mechanically activated Piezo1 maintains cardiovascular health by regulating vascular development and bone formation, whereas Piezo2 plays an essential role in fine touch perception and proprioception, as evidenced by animal models and human genetic studies (Sun et al., 2025; Taberner et al., 2019). Under pathological conditions, however, Piezo1 acts as a pivotal mediator coordinating inflammatory responses, endothelial barrier dysfunction, thrombosis, and erythrocyte dysfunction in turbulent and high-pressure environments, thereby occupying a central position in the pathogenesis of atherosclerosis; Piezo2 primarily participates in mechanical nociception and respiratory reflex regulation, findings that derive primarily from animal models with limited human validation (Shinge et al., 2022). Both channels exhibit characteristic rapid inactivation kinetics, whereby the piezoelectric current amplitude decays over time despite sustained mechanical stimulation, a property demonstrated in *ex vivo* and animal studies (Coste et al., 2010). Notably, the relative contribution of Piezo1 versus Piezo2 to post stroke cardiac pathology has not been directly compared, and no studies have examined whether stroke induced changes in pulse wave morphology differentially activate these channels.

These channels are extensively expressed in vascular endothelial cells, sensory neurons, and multiple cell types within the central nervous system. They convert mechanical signals such as blood pressure fluctuations and vascular wall shear stress into electrochemical signals. This process initiates subsequent physiological or pathological cascades, as shown in animal and *ex vivo* studies (Valenza et al., 2025).

In normal physiological regulation, baroreceptors in the carotid sinus and aortic arch perceive real-time blood pressure changes via Piezo1 and Piezo2 proteins. These signals are transmitted through the glossopharyngeal and vagus nerves to the nucleus tractus solitarius in the medulla oblongata. This process forms a complete baroreceptor reflex arc, which precisely balances sympathetic and vagal tones to maintain blood pressure homeostasis, a mechanism that has been elucidated primarily through animal models with supporting human physiological data (Zeng et al., 2018).

Acute ischemic stroke (AIS) disrupts this refined regulatory mechanism. Baroreflex arc dysfunction leads to dramatic blood pressure fluctuations and impaired heart rate control, consequently predisposing to arrhythmias or hypoperfusion injury, as documented in both human studies and animal models (Tsai et al., 2019). Simultaneously, AIS impairs cerebral autoregulation, rendering the brain more vulnerable to cardiogenic hypoperfusion or acute blood pressure variations, potentially resulting in critically compensated cerebral blood flow states and even secondary ischemic brain damage, a phenomenon observed in human physiological studies.

Emerging research further elucidates a direct dialogue mechanism in cardiocerebral mechanical coupling: the pulse waves generated by left ventricular ejection propagate through the arterial tree to cerebral microvessels. The mechanical energy they carry not only regulates local cerebral blood flow but may also directly modulate neuronal excitability and network activity by activating Piezo1 and Piezo2 mechanosensitive channels in cerebrovascular pericytes, astrocytes, and other neurovascular unit components. This interaction, occurring on the timescale of cardiac cycles, potentially represents the most direct physical communication mechanism between the heart and brain, though evidence for this direct coupling in humans remains indirect and largely extrapolated from *ex vivo* and animal studies (Choi et al., 2024). However, evidence for this direct mechanical neuronal coupling in humans remains indirect, largely extrapolated from *ex vivo* and animal studies. It is speculated that stroke-induced changes in pulse wave morphology may reach sufficient magnitude to significantly alter neuronal activity in the human brain, though this has not been directly demonstrated.

Notably, Jammal Salameh et al. (2024) recently identified a distinct neuronal subpopulation in the olfactory bulb that senses heartbeat-induced blood pressure changes, with their excitability modulated by mechanosensitive channels in animal models. This suggests that the brain can directly regulate neural activity via cardiac rhythm, revealing a rapid communication pathway along the body-brain axis. Furthermore, a groundbreaking study by the Cameron S. McAlpine team, published in Nature, revealed that ischemic heart injury promotes the infiltration of monocytes into the brain, leading to sleep disturbances. Conversely, sleep deprivation can exacerbate cardiac inflammation by promoting the aberrant accumulation of immune cells in the heart (Huynh et al., 2024). This establishes a neuroimmune-mediated vicious cycle between the heart and the brain. These findings, while compelling, have not yet been replicated in ischemic stroke models, and their relevance to human post stroke brain-heart crosstalk remains to be established.

### 2.3 HPA axis and neuroendocrine activation

The hypothalamic-pituitary-adrenal (HPA) axis, comprising the hypothalamus, pituitary gland, and adrenal glands, orchestrates the body’s adaptive response to stress (Joseph and Whirledge, 2017). Dysregulation of this neuroendocrine system frequently underlies severe stroke outcomes. Following an ischemic event, HPA axis activation triggers the release of cortisol and pro-inflammatory cytokines such as IL-6 and TNF-α. These mediators not only exacerbate cardiac injury but also compromise blood-brain barrier integrity, thereby amplifying neuroinflammation and secondary brain damage (Datta et al., 2023).

This stress-induced neuroendocrine and inflammatory response also constitutes a significant biological foundation for the development of post-stroke depression (PSD) (Zhou et al., 2022). PSD involves a complex interplay of psychological and biochemical mechanisms, where anxiety, fear, and stress significantly correlate with neurological deficits. A substantial proportion of patients experience early-onset depression directly linked to the severity of functional impairment, while others develop delayed depressive episodes (Zhou et al., 2022). Notably, emerging arrhythmias following stroke, particularly newly identified or symptomatic cases, may serve as early predictors for post-stroke depression. Successful management of these cardiac rhythm abnormalities has been associated with reduced prevalence and severity of depressive symptoms (Xu et al., 2024). Despite these associations, the causal direction remains ambiguous: it remains to be determined whether arrhythmia directly contributes to depression via reduced cerebral perfusion and altered autonomic input to limbic structures, or whether both arrhythmia and depression are independent consequences of shared neuroanatomical damage. Longitudinal studies with repeated ambulatory monitoring are needed to resolve this question.

### 2.4 Neuroinflammation and immune response

The activation of microglia and astrocytes plays a central role in post-stroke neuroinflammation, as evidenced by both human post-mortem studies and animal models. Concurrently, systemic inflammatory responses, marked by elevated levels of IL-1β and CRP, can promote atherosclerotic plaque instability, thereby increasing the risk of myocardial infarction (Jørgensen et al., 2023; Zha et al., 2021). Further evidence from diabetic mouse models indicates that NLRP3 inflammasome activation exacerbates cardiac dysfunction following ischemic stroke (Lin et al., 2020).

However, the translatability of these findings is limited by the fact that most mouse models lack the chronic atherosclerosis and cardiovascular risk factors that characterize human stroke patients. Whether NLRP3 inhibition would be beneficial or harmful in aged comorbid human populations remains unknown, as preclinical studies typically use young healthy animals.

Notably, catecholamine-stimulated blood monocytes produce interleukin-10 (IL-10), exerting immunosuppressive effects (Suberville et al., 1996). Moreover, the activation of the hypothalamic-pituitary-adrenal (HPA) axis by increased intracerebral inflammatory cytokines constitutes a crucial mechanism through which the central nervous system induces systemic immune suppression (Catania et al., 2009).

Acute brain injury triggers a sterile systemic inflammatory response (Denes et al., 2010). While modulation of neuroinflammatory pathways has been identified as a promising therapeutic strategy for protecting the ischemic brain (Li P. et al., 2024), stroke exerts profound effects not only on local neuroinflammation but also on systemic immunity (Simats and Liesz, 2022). During the hyperacute phase post-stroke, the peripheral immune system becomes rapidly activated in response to cerebral injury. This initial systemic activation is followed by an immunosuppressive state characterized by immune cell loss and functional unresponsiveness (Courties et al., 2015). In the later chronic phase, a third, less understood stage emerges, featuring persistent low-grade residual inflammation that may adversely influence long-term patient outcomes (Stanne et al., 2022). It remains to be determined whether this chronic low-grade inflammation represents a therapeutic target or merely an epiphenomenon; prospective interventional trials targeting inflammation months after stroke are lacking.

Microglial and astrocyte activation plays a central role in post-stroke neuroinflammation. The pro-inflammatory polarization of microglia during later injury phases may exacerbate ischemic damage. Their secretion of inflammatory factors further contributes to this pathology. Modulating microglial polarization toward an anti-inflammatory phenotype therefore represents a therapeutic strategy. However, these findings derive primarily from animal models with limited human validation (Shui et al., 2024).

Systemic elevations of IL-1ß and ATP levels following stroke correlate with severe cardiac impairment, including systolic dysfunction, increased oxidative stress, and myocardial inflammation (Simats et al., 2024b). Neutralizing IL-1ß or blocking pro-inflammatory monocyte trafficking with CCR2/5 inhibitors has been shown to prevent post-stroke cardiac dysfunction in animal models (Simats et al., 2024b). Critically, these promising results come exclusively from animal studies. The only large-scale human trial of interleukin 1 beta inhibition in stroke, the SCIL STROKE trial, was stopped early and did not specifically examine cardiac outcomes. Whether these experimental therapies can be safely and effectively translated to human stroke patients with cardiac complications remains an open question.

Concurrently, neuroinflammation can trigger sympathetic overactivation accompanied by cardiac injury (Jiang et al., 2024).

Catecholamine-stimulated blood monocytes produce interleukin-10 (IL-10), exerting immunosuppressive effects (Suberville et al., 1996). Activation of the hypothalamic-pituitary-adrenal (HPA) axis by increased cerebral inflammatory cytokines constitutes a key mechanism of central nervous system induced immune suppression (Catania et al., 2009; Chamorro et al., 2012). While this adaptation aims to limit excessive inflammatory damage, it significantly increases susceptibility to infections (Iadecola and Anrather, 2025). Secondary infections such as pneumonia and urinary tract infections serve as important clinical triggers for acute heart failure. This establishes an indirect injury pathway in which brain damage leads to immunosuppression, which then predisposes to infection, and subsequently increases cardiac load, ultimately resulting in cardiac dysfunction. The quantitative contribution of this indirect pathway relative to direct catecholamine mediated cardiotoxicity has not been determined.

### 2.5 Gut-brain-heart axis

Stroke induces systemic alterations beyond cerebral damage, including gastrointestinal dysfunction. Substantial evidence now supports the involvement of gut microbiota in stroke progression and recovery, with supporting data from both human and animal studies (Peh et al., 2022). Restoring gut microbial balance has emerged as a promising approach for myocardial infarction treatment (Chen et al., 2025), though evidence remains primarily from animal models with emerging human data.

Gut dysbiosis elevates thrombotic risk, particularly through increased trimethylamine N-oxide (TMAO) levels (Roberts et al., 2018). TMAO originates from microbial metabolism of dietary nutrients found in dairy products, eggs, and meat (Koeth et al., 2013). Elevated plasma TMAO concentrations predict major adverse cardiovascular events (Xu et al., 2022), and demonstrate graded correlation with subsequent cardiovascular risk in ischemic stroke patients (Haghikia et al., 2018). However, several critical limitations must be acknowledged. First, it is speculated that trimethylamine N-oxide elevation may be a consequence rather than a cause of post-stroke gut dysbiosis (Zhu et al., 2016). Second, it remains to be determined whether trimethylamine N-oxide lowering interventions such as dietary modification and microbiota transplantation can reduce post-stroke cardiac complications in humans (Heianza et al., 2017). Third, it is speculated that TMAO may be one of several microbiota-derived metabolites contributing to post-stroke cardiac risk, though the relative contribution of other metabolites including short-chain fatty acids and bile acids has not been established (Peh et al., 2022).

The clinical relevance of gut microbiota alterations is further supported by evidence linking specific metabolites to distinct outcomes. Elevated TMAO levels have been associated with increased risk of major adverse cardiovascular events after stroke, including recurrent stroke, myocardial infarction, and cardiovascular death (Frenger et al., 2026), though this association appears to vary across stroke etiologies and populations (Xu et al., 2022). With respect to functional outcomes, data remain conflicting; some studies report positive associations with unfavorable outcomes, while others find limited clinical significance. Beyond TMAO, short-chain fatty acids and lipopolysaccharide are emerging as potential mediators of post-stroke outcomes through modulation of intestinal barrier function and systemic inflammation (Peh et al., 2022).

Post-stroke intestinal barrier impairment facilitates bacterial translocation, amplifying systemic inflammation and cardiovascular complications (Wen and Wong, 2017). Recent preclinical and clinical studies further implicate gut microbiota disruption in atrial fibrillation pathogenesis (Gawałko et al., 2022).

These interconnected mechanisms form a self-perpetuating pathological network. Autonomic nervous system hyperactivity and systemic inflammation act as direct mediators of cardiac injury, while impaired cardiocerebral mechanical coupling and HPA axis activation establish the conditions for disease progression. The convergence of these pathways manifests as characteristic clinical syndromes of brain-heart interaction.

The intricate interplay among autonomic dysregulation, HPA axis activation, neuroinflammation, immunosuppression, mechanical coupling, and gut microbiota disruption forms a self-perpetuating pathological network following ischemic stroke. These interconnected mechanisms do not operate in isolation but rather synergistically contribute to both cardiac complications and secondary brain injury. A concise summary of these core mechanisms is presented in Table 1.

**TABLE 1:** Summary of core mechanisms in the brain-heart axis.

Mechanism category	Key mediators/structures	Primary effects	Clinical consequences	Key supporting studies
Autonomic dysregulation	Insular cortex, sympathetic nervous system, catecholamines	Myocardial calcium overload, contraction band necrosis	Takotsubo syndrome, arrhythmias	Colombari et al., 2025; Hachinski et al., 1986; Kang et al., 2024
HPA axis activation	Cortisol, IL-6, TNF-a	Blood-brain barrier disruption, neuroinflammation	Secondary brain injury, post-stroke depression (PSD)	Datta et al., 2023; Joseph and Whirledge, 2017
Neuroinflammation	Microglia, IL-1ß, NLRP3 inflammasome	Myocardial dysfunction	Cardiac dysfunction	Jørgensen et al., 2023; Lin et al., 2020; Zha et al., 2021
Immunosuppression	IL-10, HPA axis	Increased susceptibility to infections	Pneumonia, heart failure trigger	Catania et al., 2009; Suberville et al., 1996
Mechanical coupling	Piezo1/2, baroreceptors	Blood pressure regulation, neuronal excitability	Hypoperfusion, arrhythmias	Coste et al., 2010; Ince et al., 2020; Shinge et al., 2022
Gut-brain-heart axis	TMAO, gut microbiota	Thrombosis, inflammatory amplification	Major adverse cardiovascular events (MACE), atrial fibrillation	Peh et al., 2022; Roberts et al., 2018; Xu et al., 2022; Zhu et al., 2016

### 2.6 Crosstalk between mechanisms

The pathways described above do not operate in isolation but rather engage in complex bidirectional crosstalk that collectively constitutes a self-reinforcing pathological network. This crosstalk not only amplifies the effects of individual pathways but, more importantly, produces systemic pathological consequences that cannot be explained by any single pathway alone.

Autonomic dysregulation and neuroinflammation form a particularly prominent feed-forward loop. Sympathetic overactivation directly promotes the release of pro-inflammatory cytokines such as IL-1ß and TNF-a from immune cells via ß-adrenergic receptor signaling (Barkhudaryan et al., 2025), while central and peripheral inflammatory mediators in turn sensitize autonomic centers including the insular cortex, anterior cingulate cortex, and hypothalamus, further exacerbating sympathetic outflow (Zhu et al., 2022). This positive feedback loop simultaneously amplifies cardiac injury and cerebral inflammation, explaining why the degree of post-stroke autonomic dysfunction is closely correlated with inflammatory marker levels and the severity of cardiac complications (Pongratz and Straub, 2014).

Neuroinflammation and the HPA axis exhibit a similarly bidirectional relationship (Li P. et al., 2026). Pro-inflammatory cytokines stimulate the hypothalamus to release corticotropin-releasing hormone, activating the HPA axis and promoting cortisol secretion (Zhou et al., 2022). Cortisol, in turn, modulates immune cell differentiation and function through glucocorticoid receptors, limiting excessive inflammation while simultaneously increasing susceptibility to infection (Urra et al., 2009). This interaction provides a mechanistic explanation for the observed association between post-stroke infection and cardiac complications.

Mechanical coupling intersects with both autonomic and inflammatory pathways. Baroreceptor dysfunction leads to unopposed blood pressure fluctuations, and the resulting hemodynamic instability can trigger inflammatory responses through shear stress-mediated endothelial activation (Wang et al., 2025). Conversely, inflammatory cytokines such as TNF-α can impair baroreceptor sensitivity, establishing a vicious cycle between hemodynamic and inflammatory dysfunction (Lataro et al., 2024). The extensive expression of Piezo channels in vascular endothelial cells, sensory neurons, and the central nervous system positions them as critical nodes for converting mechanical signals into electrochemical signals (Beech and Kalli, 2019), though the specific contribution of this pathway to post-stroke brain-heart interactions requires further validation.

The gut-brain-heart axis serves as a central hub integrating multiple pathways, linking gut dysbiosis with systemic inflammation, autonomic function, and cardiac injury. Increased intestinal permeability and dysbiosis after stroke promote bacterial translocation and TMAO production, amplifying systemic inflammatory burden (Granados-Martinez et al., 2024). Sympathetic activation further impairs intestinal barrier function, establishing a complex loop involving the gut, brain, heart, and autonomic nervous system (Li Z. et al., 2024).

These interconnections have clear translational implications. First, they suggest that single-target interventions may be insufficient to disrupt the pathological network, and that combination strategies warrant investigation. Second, the temporal profiles of different pathways suggest that therapeutic windows may differ: autonomic modulation is most critical during the hyperacute to acute phase, anti-inflammatory interventions may play a greater role in the subacute phase, and gut microbiota modulation may hold potential across the entire recovery period. Understanding these interconnections is essential for developing rational, mechanism-based combination therapeutic strategies for stroke-heart syndrome.

## 3 Clinical complications of stroke-related brain-heart interactions

### 3.1 Cardiac manifestations

Stroke-induced brain-heart interactions produce diverse clinical presentations (Wang et al., 2024). Takotsubo syndrome affects a notable proportion of severe stroke cases, featuring transient left ventricular dysfunction with characteristic apical ballooning and reduced ejection fraction (Stengl et al., 2024). Arrhythmias represent another common complication, with atrial fibrillation occurring in a substantial subset of patients. QT prolongation and ventricular arrhythmias also frequently appear, primarily linked to insular cortex damage and sympathetic overactivation (Hatab et al., 2025). Left ventricular diastolic dysfunction is detectable in a considerable number of stroke patients and correlates significantly with poor prognosis (Park et al., 2016).

A significant diagnostic challenge deserves emphasis: distinguishing Takotsubo syndrome from acute myocardial infarction in the acute stroke setting is often impossible without coronary angiography, which carries additional risks in stroke patients. Consequently, the reported incidence of Takotsubo syndrome may substantially underestimate its true prevalence, as many cases are misclassified as non ST elevation myocardial infarction or cardiomyopathy of unknown etiology. Furthermore, the optimal management of atrial fibrillation detected after stroke versus known pre stroke atrial fibrillation remains controversial, with conflicting evidence regarding whether atrial fibrillation detected after stroke represents a distinct entity with lower embolic risk.

These complications vary considerably in their clinical features, underlying mechanisms, and prognostic implications. A concise summary is presented in Table 2.

**TABLE 2:** Cardiac complications following stroke: types and characteristics.

Complication	Key features	Mechanisms	Prognostic implications
Takotsubo syndrome	Apical ballooning, reduced LVEF	Catecholamine toxicity, sympathetic storm	Reversible acutely, elevated long-term MACE risk
Atrial fibrillation	New-onset or paroxysmal	Autonomic imbalance, systemic inflammation	Increased embolic risk, predictor of PSD
QT prolongation	Susceptibility to malignant arrhythmias	Insular cortex injury, sympathetic overactivation	Predisposes to ventricular fibrillation, sudden death
Left ventricular diastolic dysfunction	Impaired filling, elevated filling pressure	Autonomic dysregulation, neuroinflammation	Significantly associated with adverse outcomes

### 3.2 Secondary brain injury

Cardiac dysfunction can exacerbate cerebral damage through multiple pathways. Reduced cardiac output decreases cerebral blood flow, particularly in patients with impaired autoregulation, leading to cerebral hypoperfusion that may trigger watershed infarction or silent ischemia (Meng et al., 2015). Left ventricular dysfunction elevates embolic risk, with microthrombi causing covert cerebral infarction or transient ischemic attacks, especially in patients with atrial fibrillation or ventricular thrombi (McNamara et al., 2024).

Systemic inflammatory mediators cross the compromised blood-brain barrier, activating microglia and perpetuating neuroinflammation. This establishes a self-reinforcing cycle of neuronal damage and synaptic impairment (Candelario-Jalil et al., 2022).

Additionally, cardiogenic edema and hypoxic states intensify cerebral metabolic disturbance and oxidative stress, expanding the ischemic penumbra and worsening neurological deficits (Zou et al., 2025). A major knowledge gap exists regarding the time course of secondary brain injury from cardiac dysfunction: is there a critical window during which aggressive cardiac support could salvage threatened brain tissue? Current guidelines provide no specific recommendations on this point. These interconnected mechanisms illustrate the complex clinical spectrum of post-stroke brain-heart syndrome, emphasizing critical multi-system interactions.

## 4 Therapeutic and management strategies

A fundamental limitation of current therapeutic approaches must be acknowledged at the outset: virtually no management strategies for post stroke brain heart complications have been specifically validated in randomized controlled trials enrolling stroke populations. The recommendations summarized below are therefore largely extrapolated from general cardiology or neurocritical care guidelines, with the exception of blood pressure management in acute ischemic stroke for which stroke specific trial data exist.

### 4.1 Monitoring and risk assessment

High-risk AIS patients should receive ICU care with continuous hemodynamic monitoring for at least 72 h. This enables prompt detection of malignant arrhythmias and QTc prolongation (Battaglini et al., 2020). Serial measurements of high-sensitivity cardiac troponin and B-type natriuretic peptide help identify acute myocardial injury and early heart failure. Their dynamic changes offer significant prognostic value (Goryacheva et al., 2022; Haller et al., 2024). However, the optimal sampling frequency and the threshold values that should trigger intervention in the stroke population have not been established;

Echocardiography should be performed within 48 h of admission. Assessment should cover left ventricular function, regional wall motion abnormalities, apical ballooning suggestive of Takotsubo syndrome, and intracardiac thrombi. These findings guide anticoagulation and cardiac protection strategies. Echocardiographic evidence also supports the concept of central autonomic network damage causing cardiac impairment (Winder et al., 2023). Combined brain-heart MRI provides early diagnosis of dual pathology. In complex cases, multimodal imaging integrating brain MRI and cardiac MR comprehensively evaluates brain-heart interaction damage (Markousis-Mavrogenis et al., 2022). Cardiac MRI faces practical limitations in acute stroke patients. These include prolonged scan time, need for patient cooperation, and contrast administration concerns. Therefore, its widespread application remains challenging.

### 4.2 Pharmacological interventions

Treatment strategies include β-blockers, anti-inflammatory therapy, anticoagulation, and neuroendocrine modulation (Chen et al., 2017). Selective β1-blockers may be initiated after acute stroke stabilization. They suppress sympathetic overactivation and reduce catecholamine toxicity. This may decrease malignant arrhythmias and Takotsubo syndrome (Lee et al., 2025). Careful monitoring is essential to avoid cerebral hypoperfusion from hypotension or bradycardia.

However, several critical uncertainties exist. First, the optimal timing of initiation has not been defined. Early initiation risks hypotension and worsened cerebral ischemia, while delayed initiation may miss the therapeutic window. Second, the appropriate patient selection criteria remain unknown. It is unclear whether all large hemisphere strokes should receive beta blockers or only those with evidence of cardiac injury. Third, the optimal duration of therapy has not been studied. Fourth, no randomized controlled trial has specifically examined beta blockers for this indication, representing a major evidence gap.

Targeted anti-inflammatory approaches offer new directions. Interleukin 1 beta inhibitors such as canakinumab demonstrated cardiovascular protection in the CANTOS trial, reducing myocardial infarction and stroke recurrence (Dhorepatil et al., 2019). However, the CANTOS trial enrolled patients with prior myocardial infarction and elevated high sensitivity C reactive protein, not acute stroke. Whether canakinumab is safe or effective in the acute post stroke setting, particularly regarding infection risk given stroke induced immunosuppression, has not been tested. However, their safety and efficacy in stroke patients with cardiac complications need specific investigation. Emerging evidence supports colchicine in coronary disease, though data in stroke populations remains lacking (Bouabdallaoui and Tardif, 2022). No anti-inflammatory agent has completed a phase 3 trial specifically for post stroke cardiac complications.

Patients with atrial fibrillation or ventricular thrombi require individualized anticoagulation based on CHA_2_DS_2_-VASc and HAS-BLED scores. NOACs are generally preferred over warfarin due to better safety profiles. However, in large cerebral infarctions or high bleeding risk patients, initiation should be delayed with close imaging monitoring (Kleindorfer et al., 2021). The optimal timing for anticoagulation initiation after stroke in patients with cardiac thrombus represents a persistent clinical dilemma: early initiation reduces embolic recurrence risk but increases hemorrhagic transformation risk. Current guidelines acknowledge this equipoise and call for individualized decision making, but validated risk stratification tools are lacking.

Mineralocorticoid receptor antagonists such as spironolactone and SGLT2 inhibitors including empagliflozin show potential for dual cardiocerebral protection in heart failure. Their benefits may involve edema reduction, metabolic improvement, and antifibrotic effects. Research in stroke-heart syndrome populations remains preliminary (Packer et al., 2020). No studies have specifically examined SGLT2 inhibitors in patients with stroke induced cardiac dysfunction; current recommendations are extrapolated from heart failure with reduced ejection fraction trials that excluded acute stroke patients.

A summary of these pharmacological strategies, including their potential benefits and clinical precautions, is presented in Table 3.

**TABLE 3:** Pharmacological interventions and clinical considerations.

Drug class	Representative agents	Benefits	Precautions
β-Blockers	Bisoprolol, metoprolol	Sympathetic inhibition, reduced catecholamine toxicity	Hypotension, bradycardia
Anti-inflammatory agents	Canakinumab, colchicine	Reduced cardiovascular events	Limited evidence in stroke
Anticoagulants	NOACs, warfarin	Embolism prevention	Delay if large infarct or high bleeding risk
SGLT2 Inhibitors	Empagliflozin	Cardio-renal protection, anti-fibrosis	Limited data in stroke-heart syndrome
Mineralocorticoid receptor antagonists	Spironolactone	Edema reduction, anti-fibrosis	Monitor potassium, renal function

To provide clearer guidance on biomarker-driven therapy selection, we summarize key biomarkers and their therapeutic implications in Table 4. This framework links specific biomarkers to the pathways they reflect and the interventions they may guide, including troponin for beta-blocker consideration, natriuretic peptides for diuretic and afterload management, inflammatory markers for anti-inflammatory approaches, and TMAO for potential microbiota-targeted strategies.

**TABLE 4:** Biomarkers and therapeutic implications.

Biomarker	Pathway reflected	Therapeutic implication
High-sensitivity cardiac troponin	Myocardial injury	Consider beta-blocker therapy
BNP/NT-proBNP	Hemodynamic stress	Guide diuretics, afterload reduction
hs-CRP, IL-6	Systemic inflammation	Consider anti-inflammatory approaches
TMAO	Gut dysbiosis	Potential target for microbiota modulation; remains investigational

### 4.3 Blood pressure management

Blood pressure control requires balancing cerebral perfusion and cardiac afterload reduction. According to AHA/ASA guidelines, patients without revascularization should start antihypertensive therapy when systolic pressure consistently exceeds 220 mmHg or diastolic pressure exceeds 120 mmHg. The initial 24-h reduction should not exceed 15% (Jones et al., 2025).

In patients with acute heart failure, ACS, or Takotsubo syndrome, management must consider both cerebral and cardiac needs. Drugs without reflex tachycardia such as labetalol or urapidil are preferred. Short-acting nifedipine should be avoided due to risk of abrupt hypotension (Peixoto, 2019).

Long-term management favors ACEIs/ARBs for their cardiovascular and potential neurological benefits. The general target is <130/80 mmHg, though this should be relaxed for patients with severe bilateral carotid stenosis or impaired cerebral autoregulation (Kleindorfer et al., 2021). Notably, the optimal blood pressure target for patients with concurrent acute ischemic stroke and acute cardiac dysfunction has never been specifically studied; current recommendations represent expert consensus only.

## 5 Future research directions

The clinical burden of post-stroke cardiac complications has been quantified in several large-scale cohort studies and registry analyses. A comparative summary of key clinical studies is presented in Table 5.

**TABLE 5:** Comparative summary of key clinical studies on post-stroke cardiac complications.

References	Population	Key findings
Buckley et al., 2022	365,383 ischemic stroke	30-day incidence: ACS 11.1%, AF 8.8%, HF 6.4%; 5-year mortality elevated for all (OR 1.45–2.08)
Jang et al., 2026	3,376,606 stroke	TTS incidence 0.18%; TTS-complicated mortality 21% vs. 7.8% (*p* < 0.001); TTS more frequent in females and hemorrhagic stroke
Ishiguchi et al., 2025	12,482 ischemic stroke	Five SHS phenotypes identified; cardiac comorbidities or elderly+AF profiles had highest risk (aHR 2.01 and 1.26)

To move beyond the descriptive limitations noted in the critique of this review and the broader field, we propose a prioritized research agenda that transitions from observational synthesis to hypothesis driven investigation. This agenda is organized across four temporal horizons with specific milestones.

Over the immediate 1–2 year horizon, efforts must focus on establishing causal relationships in humans. Prospective cohort studies with serial biosampling every 6–12 h for the first 72 h after stroke and then daily thereafter are needed to define the temporal dynamics of catecholamines, cytokines, and cardiac biomarkers. Such studies would enable determination of whether autonomic dysfunction precedes or follows cardiac injury, a distinction with direct therapeutic implications. Concurrently, multicenter registries with standardized phenotyping should be established to quantify the relative contribution of each mechanism including autonomic, inflammatory, mechanical, and gut derived pathways using mediation analysis in large patient cohorts. A third priority in this timeframe is validation of atrial fibrillation detected after stroke as a distinct entity through prospective monitoring comparing embolic event rates between atrial fibrillation detected after stroke and known pre stroke atrial fibrillation, which would directly inform anticoagulation strategies.

In the 2–5 year timeframe, interventional trials become the central priority. A phase 2 randomized controlled trial of early beta blocker therapy should be conducted in patients with large hemisphere stroke and evidence of cardiac injury including elevated troponin or echocardiographic wall motion abnormality. The co primary outcomes should include safety endpoints such as hypotension and worsened neurologic deficit as well as efficacy endpoints including arrhythmia reduction and troponin attenuation. Parallel to this, a pilot trial of gut microbiota modulation such as dietary intervention or probiotic administration should measure trimethylamine N oxide levels and exploratory cardiac outcomes to establish feasibility and effect size for a definitive trial. These initial trials are essential to generate the preliminary data needed for larger phase 3 studies.

Over the 3–7 year horizon, mechanistic deep dive studies should take precedence. Single cell and spatial transcriptomic analyses of post stroke human cardiac tissue obtained from endomyocardial biopsy or autopsy would identify immune cell infiltration patterns and Piezo channel expression while enabling direct comparison with animal model findings. This approach would address the critical question of whether mechanisms identified in rodent models truly operate in human stroke. Complementing these tissue studies, the development of human induced pluripotent stem cell derived cardiomyocyte models exposed to post stroke sera or catecholamine surges would allow identification of genetic modifiers of susceptibility to catecholamine induced cardiotoxicity, potentially enabling future risk stratification.

For the long term 5–10 year horizon, precision medicine implementation represents the ultimate goal. Integration of multimodal biomarkers including genetic risk scores, circulating microRNAs, and imaging phenotypes into a validated predictive algorithm for post stroke cardiac complications should be tested in prospective validation cohorts. Once validated, such algorithms could guide clinical decision making by identifying high risk patients who would most benefit from targeted interventions. Furthermore, platform trial designs allowing simultaneous testing of multiple mechanism targeted interventions including beta blockers, interleukin 1 inhibitors, trimethylamine N oxide lowering strategies, and vagal nerve stimulation with adaptive allocation based on patient specific biomarker profiles would accelerate the evaluation of emerging therapies.

For the long-term 5–10 year horizon, precision medicine implementation represents the ultimate goal. Integration of multimodal biomarkers including genetic risk scores, circulating microRNAs, and imaging phenotypes into a validated predictive algorithm for post-stroke cardiac complications should be tested in prospective validation cohorts. Once validated, such algorithms could guide clinical decision making by identifying high-risk patients who would most benefit from targeted interventions. Furthermore, the development of systems biology and network-based quantitative frameworks that integrate multi-omics data with clinical parameters holds substantial promise for identifying critical regulatory nodes and predicting individual patient trajectories. Such approaches would enable mechanistic simulation of brain-heart crosstalk and facilitate the identification of optimal therapeutic targets and intervention timing.

Across all these horizons, several specific techniques and technologies merit prioritization. Single cell sequencing and spatial transcriptomics will reveal immune memory formation and neural cardiac circuit remodeling after stroke. Investigation of mitochondrial signaling between organs may identify entirely new therapeutic targets. Neuromodulation techniques including vagal nerve stimulation and stellate ganglion blockade offer promise for restoring autonomic balance and controlling inflammation but require rigorous testing in stroke specific populations. Multi omics integration encompassing genomics, proteomics, metabolomics, and metagenomics is needed for biomarker discovery. Above all, establishing dedicated well-phenotyped cohorts for brain heart diseases is essential to provide the infrastructure for all these proposed investigations. Without such cohorts, progress will remain slow and fragmented.

## 6 Limitations of current evidence

Several important limitations must be acknowledged when interpreting the evidence summarized in this review. These constraints span three interconnected domains and collectively highlight the gaps that must be addressed to advance the field.

Most mechanistic insights derive from animal models, particularly rodent middle cerebral artery occlusion. These models have provided fundamental understanding of autonomic dysregulation, neuroinflammation, and gut-brain-heart interactions. However, their translatability to human stroke is constrained. Interspecies differences in cardiovascular physiology, immune responses, and gut microbiota composition limit the applicability of these findings. Furthermore, these models typically employ young, healthy animals and lack the chronic comorbidities such as hypertension, diabetes, and atherosclerosis that characterize human stroke patients. Human data remain predominantly observational, with few randomized controlled trials specifically designed to evaluate interventions targeting brain-heart interactions.

Diagnostic tools for stroke-heart syndrome remain suboptimal. Cardiac complications frequently evade detection due to atypical presentations, particularly in elderly and diabetic populations. Biomarker thresholds for intervention are extrapolated from acute coronary syndrome trials rather than validated in stroke populations. Therapeutic strategies are largely adapted from general cardiology or neurocritical care guidelines. No stroke-specific guidelines exist for managing post-stroke cardiac complications. The optimal timing, patient selection, and duration of beta-blocker therapy remain undefined. No anti-inflammatory agent has completed a phase 3 trial specifically for post-stroke cardiac complications.

Multiple causal relationships remain unresolved. It is unknown whether autonomic dysfunction is the primary driver of cardiac injury or merely an epiphenomenon of severe stroke. The temporal hierarchy of pathway activation has not been systematically established. The relative contribution of each mechanism to clinical outcomes remains undefined.

Whether TMAO elevation is causal or merely a marker of gut dysbiosis is uncertain. TMAO-lowering interventions have not been shown to improve outcomes. Individual susceptibility to stroke-heart syndrome, including the role of genetic modifiers and pre-existing cardiac reserve, remains poorly understood. Collectively, these limitations underscore the need for prospective human studies, stroke-specific randomized trials, and integration of multi-omics approaches to advance the field. Critically, addressing these gaps will require interdisciplinary collaboration among neuroscientists, cardiologists, immunologists, and clinical trialists.

## 7 Conclusion

Brain-heart crosstalk form a complex network involving neurohumoral, inflammatory, hemodynamic, and gut brain pathways. However, as critically appraised throughout this review, current understanding is limited by predominantly observational evidence, questionable translatability of animal models, absence of stroke specific randomized controlled trials for most interventions, and major unresolved knowledge gaps regarding causal relationships, temporal dynamics, and individual susceptibility. Effective management requires collaboration between neurology, cardiology, critical care, and rehabilitation.

While significant progress has been made in describing mechanisms and developing potential interventions, the field must now transition from descriptive integration to hypothesis driven interventional research. Specifically, future work should prioritize: first, prospective human studies establishing temporal causality; second, randomized controlled trials of beta blockers, anti-inflammatory agents, and gut microbiota modulation specifically in stroke heart syndrome; third, mechanistic deep dive studies using modern multi omics and human cell models; and fourth, development and validation of predictive algorithms for precision medicine. Bridging basic science and clinical practice through rigorous trials and real world studies is crucial. This will support the development of evidence based stroke specific guidelines to improve long term outcomes and quality of life for patients with brain heart conditions.

## References

[B1] Barkhudaryan A. Doehner W. Jauert N. (2025). Autonomic dysfunction after stroke: An overview of recent clinical evidence and perspectives on therapeutic management. *Clin. Auton. Res.* 35 553–563. 10.1007/s10286-025-01120-0 40131648 PMC12325444

[B2] Battaglini D. Robba C. Lopes da Silva A. Dos Santos Samary C. Leme Silva P. Dal Pizzol F. et al. (2020). Brain-heart interaction after acute ischemic stroke. *Crit. Care* 24:163. 10.1186/s13054-020-02885-8 32317013 PMC7175494

[B3] Beech D. Kalli A. (2019). Force sensing by piezo channels in cardiovascular health and disease. *Arterioscler. Thromb. Vasc. Biol.* 39 2228–2239. 10.1161/ATVBAHA.119.313348 31533470 PMC6818984

[B4] Bouabdallaoui N. Tardif J. (2022). Repurposing colchicine for heart disease. *Annu. Rev. Pharmacol. Toxicol.* 62 121–129. 10.1146/annurev-pharmtox-052120-020445 34587458

[B5] Buckley B. Harrison S. Hill A. Underhill P. Lane D. Lip G. (2022). *Stroke*-heart syndrome: Incidence and clinical outcomes of cardiac complications following stroke. *Stroke* 53 1759–1763. 10.1161/STROKEAHA.121.037316 35354300

[B6] Candelario-Jalil E. Dijkhuizen R. Magnus T. (2022). Neuroinflammation, *Stroke*, blood-brain barrier dysfunction, and imaging modalities. *Stroke* 53 1473–1486. 10.1161/STROKEAHA.122.036946 35387495 PMC9038693

[B7] Catania A. Lonati C. Sordi A. Gatti S. (2009). Detrimental consequences of brain injury on peripheral cells. *Brain Behav. Immun.* 23 877–884. 10.1016/j.bbi.2009.04.006 19394418

[B8] Celaj S. Prabhakaran S. (2019). Cardioembolic sources in patients with small single subcortical infarcts. *Neurologist* 24 56–58. 10.1097/NRL.0000000000000224 30817491

[B9] Chamorro Á Meisel A. Planas A. M. Urra X. van de Beek D. Veltkamp R. (2012). The immunology of acute stroke. *Nat. Rev. Neurol.* 8 401–410. 10.1038/nrneurol.2012.98 22664787

[B10] Chen J. Zhu T. Yang J. Shen M. Wang D. Gu B. et al. (2025). Geniposide protects against myocardial infarction injury via the restoration in gut microbiota and gut-brain axis. *J. Cell. Mol. Med.* 29:e70406. 10.1111/jcmm.70406 39910683 PMC11798748

[B11] Chen Z. Venkat P. Seyfried D. Chopp M. Yan T. Chen J. (2017). Brain-heart interaction: Cardiac complications after stroke. *Circ. Res.* 121 451–468. 10.1161/CIRCRESAHA.117.311170 28775014 PMC5553569

[B12] Choi S. E. Tsang W. Huang Y. Lau K. Bucci T. Lam S. et al. (2024). Stroke-heart syndrome: A population-based cohort study and cluster analysis. *Eur Heart J.* 45:ehae666.2320. 10.1093/eurheartj/ehae666.2320

[B13] Colombari E. Biancardi V. Colombari D. Katayama P. Medeiros F. Aitken A. et al. (2025). Hypertension, blood-brain barrier disruption and changes in intracranial pressure. *J. Physiol.* 603 2245–2261. 10.1113/JP285058 40163552 PMC12453937

[B14] Coste B. Mathur J. Schmidt M. Earley T. Ranade S. Petrus M. et al. (2010). Piezo1 and Piezo2 are essential components of distinct mechanically activated cation channels. *Science* 330 55–60. 10.1126/science.1193270 20813920 PMC3062430

[B15] Courties G. Herisson F. Sager H. Heidt T. Ye Y. Wei Y. et al. (2015). Ischemic stroke activates hematopoietic bone marrow stem cells. *Circ. Res.* 116 407–417. 10.1161/CIRCRESAHA.116.305207 25362208 PMC4312511

[B16] Datta A. Saha C. Godse P. Sharma M. Sarmah D. Bhattacharya P. (2023). Neuroendocrine regulation in stroke. *Trends Endocrinol. Metab.* 34 260–277. 10.1016/j.tem.2023.02.005 36922255

[B17] Denes A. Thornton P. Rothwell N. Allan S. (2010). Inflammation and brain injury: Acute cerebral ischaemia, peripheral and central inflammation. *Brain Behav. Immun.* 24 708–723. 10.1016/j.bbi.2009.09.010 19770034

[B18] Dhorepatil A. Ball S. Ghosh R. Kondapaneni M. Lavie C. (2019). Canakinumab: Promises and future in cardiometabolic diseases and malignancy. *Am. J. Med.* 132 312–324. 10.1016/j.amjmed.2018.10.013 30832770

[B19] Feigin V. Brainin M. Norrving B. Martins S. Pandian J. Lindsay P. et al. (2025). World stroke organization: Global stroke fact sheet 2025. *Int. J. Stroke* 20 132–144. 10.1177/17474930241308142 39635884 PMC11786524

[B20] Frenger J. Jeker B. Arnold M. Grosse G. Pokorny T. Westphal L. et al. (2026). Trimethylamine N-oxide (TMAO) for risk stratification after acute ischemic stroke: Results from the BIOSIGNAL cohort study. *Eur. Stroke J.* 11:23969873251366192. 10.1093/esj/23969873251366192 40916446 PMC12417456

[B21] Gawałko M. Agbaedeng T. Saljic A. Müller D. Wilck N. Schnabel R. et al. (2022). Gut microbiota, dysbiosis and atrial fibrillation. Arrhythmogenic mechanisms and potential clinical implications. *Cardiovasc. Res.* 118 2415–2427. 10.1093/cvr/cvab292 34550344 PMC9400433

[B22] GBD 2021 Stroke Risk Factor Collaborators (2024). Global, regional, and national burden of stroke and its risk factors, 1990-2021: A systematic analysis for the Global Burden of Disease Study 2021. *Lancet Neurol.* 23 973–1003. 10.1016/S1474-4422(24)00369-7 39304265 PMC12254192

[B23] Goryacheva O. Ponomaryova T. Drozd D. Kokorina A. (2022). Heart failure biomarkers BNP and NT-proBNP detection using optical labels. *Trac-Trends Analytical Chem.* 146:116477. 10.1016/j.trac.2021.116477

[B24] Granados-Martinez C. Alfageme-Lopez N. Navarro-Oviedo M. Nieto-Vaquero C. Cuartero M. Diaz-Benito B. et al. (2024). Gut microbiota, bacterial translocation, and stroke: Current knowledge and future directions. *Biomedicines* 12:2781. 10.3390/biomedicines12122781 39767686 PMC11673227

[B25] Hachinski V. Smith K. Silver M. Gibson C. Ciriello J. (1986). Acute myocardial and plasma catecholamine changes in experimental stroke. *Stroke* 17 387–390. 10.1161/01.str.17.3.387 3715933

[B26] Haghikia A. Li X. Liman T. Bledau N. Schmidt D. Zimmermann F. et al. (2018). Gut microbiota-dependent trimethylamine N-oxide predicts risk of cardiovascular events in patients with stroke and is related to proinflammatory monocytes. *Arterioscler. Thromb. Vasc. Biol.* 38 2225–2235. 10.1161/ATVBAHA.118.311023 29976769 PMC6202215

[B27] Haller P. Jarolim P. Palazzolo M. Bellavia A. Antman E. Eikelboom J. et al. (2024). Heart failure risk assessment using biomarkers in patients with atrial fibrillation: Analysis from COMBINE-AF. *J. Am. Coll. Cardiol.* 84 1528–1540. 10.1016/j.jacc.2024.07.023 39230543

[B28] Hatab I. Kneihsl M. Arnold M. Pokorny T. Westphal L. Bicciato G. et al. (2025). Role of NT-proBNP for atrial fibrillation detection after ischemic *Stroke*: A time-dependent relationship. *Stroke* 56 1704–1713. 10.1161/STROKEAHA.124.049249 40255172

[B29] Heianza Y. Ma W. Manson J. Rexrode K. Qi L. (2017). Gut microbiota metabolites and risk of major adverse cardiovascular disease events and death: A systematic review and meta-analysis of prospective studies. *J. Am. Heart Assoc.* 6:e004947. 10.1161/JAHA.116.004947 28663251 PMC5586261

[B30] Hoad K. Jones H. Miller G. Abdul-Rahim A. Lip G. Buckley B. (2025). Stroke-heart syndrome: Incidence and clinical outcomes of cardiac complications following intracerebral haemorrhage. *Eur. Stroke J.* 10 100–107. 10.1177/23969873241264115 39080982 PMC11569547

[B31] Huynh P. Hoffmann J. Gerhardt T. Kiss M. Zuraikat F. Cohen O. et al. (2024). Myocardial infarction augments sleep to limit cardiac inflammation and damage. *Nature* 635 168–177. 10.1038/s41586-024-08100-w 39478215 PMC11998484

[B32] Iadecola C. Anrather J. (2025). The immunology of stroke and dementia. *Immunity* 58 18–39. 10.1016/j.immuni.2024.12.008 39813992 PMC11736048

[B33] Inamasu J. Sugimoto K. Watanabe E. Kato Y. Hirose Y. (2013). Effect of insular injury on autonomic functions in patients with ruptured middle cerebral artery aneurysms. *Stroke* 44 3550–3552. 10.1161/STROKEAHA.113.003099 24065710

[B34] Ince J. Banahan C. Venturini S. Alharbi M. Turner P. Oura M. et al. (2020). Acute ischemic stroke diagnosis using brain tissue pulsations. *J. Neurol. Sci.* 419:117164. 10.1016/j.jns.2020.117164 33045670

[B35] Ishiguchi H. Huang B. El-Bouri W. Lip G. Abdul-Rahim A. (2025). Stroke-heart syndrome and early mortality in patients with acute ischaemic stroke using hierarchical cluster analysis: An individual patient data pooled analysis from the VISTA database. *Eur. Stroke J.* 10 478–486. 10.1177/23969873241290440 39397359 PMC11556556

[B36] Jammal Salameh L. Bitzenhofer S. Hanganu-Opatz I. Dutschmann M. Egger V. (2024). Blood pressure pulsations modulate central neuronal activity via mechanosensitive ion channels. *Science* 383:eadk8511. 10.1126/science.adk8511 38301001

[B37] Jang S. Sposato L. Scheitz J. Choi Y. Geressu A. Tzemos N. et al. (2026). Clinical impact of Takotsubo syndrome in patients with stroke. *Heart* 10.1136/heartjnl-2025-327188 Online ahead of print. 42045052

[B38] Jiang M. Zhu Z. Zhou Z. Yan Z. Huang K. Jiang R. et al. (2024). A temperature-ultrasound sensitive nanoparticle delivery system for exploring central neuroinflammation mechanism in stroke-heart syndrome. *J. Nanobiotechnol.* 22:681. 10.1186/s12951-024-02961-z 39506743 PMC11542249

[B39] Johansen M. von Rennenberg R. Nolte C. Jensen M. Bustamante A. Katan M. (2024). Role of cardiac biomarkers in *Stroke* and cognitive impairment. *Stroke* 55 2376–2384. 10.1161/STROKEAHA.123.044143 39016019 PMC11347090

[B40] Jones D. Ferdinand K. Taler S. Johnson H. Shimbo D. Abdalla M. et al. (2025). 2025 AHA/ACC/AANP/AAPA/ABC/ACCP/ACPM/AGS/AMA/ ASPC/NMA/PCNA/SGIM guideline for the prevention, detection, evaluation and management of high blood pressure in adults: A report of the American college of cardiology/American heart association joint committee on clinical practice guidelines. *Hypertension* 82 e212–e316. 10.1161/HYP.0000000000000249 40811516

[B41] Jørgensen S. M. Lorentzen L. G. Chuang C. Y. Davies M. J. (2023). IL-1b as a potential biomarker and therapeutic target in coronary artery disease. *Eur. Heart J.* 44:ehad655.3137. 10.1093/eurheartj/ehad655.3137

[B42] Joseph D. Whirledge S. (2017). Stress and the HPA Axis: Balancing homeostasis and fertility. *Int. J. Mol. Sci.* 18:2224. 10.3390/ijms18102224 29064426 PMC5666903

[B43] Kang K. Shi K. Liu J. Li N. Wu J. Zhao X. (2024). Autonomic dysfunction and treatment strategies in intracerebral hemorrhage. *CNS Neurosci. Ther.* 30:e14544. 10.1111/cns.14544 38372446 PMC10875714

[B44] Kleindorfer D. Towfighi A. Chaturvedi S. Cockroft K. Gutierrez J. Lombardi-Hill D. et al. (2021). 2021 guideline for the prevention of *Stroke* in patients with stroke and transient ischemic attack: A guideline from the American heart association/American stroke association. *Stroke* 52 e364–e467. 10.1161/STR.0000000000000375 34024117

[B45] Koeth R. Wang Z. Levison B. Buffa J. Org E. Sheehy B. et al. (2013). Intestinal microbiota metabolism of L-carnitine, a nutrient in red meat, promotes atherosclerosis. *Nat. Med.* 19 576–585. 10.1038/nm.3145 23563705 PMC3650111

[B46] Lataro R. Brognara F. Iturriaga R. Paton J. (2024). Inflammation of some visceral sensory systems and autonomic dysfunction in cardiovascular disease. *Auton. Neurosci.* 251:103137. 10.1016/j.autneu.2023.103137 38104365

[B47] Lee K. Kim S. Guk H. Kim D. Kim B. Han M. et al. (2025). Persistent beta-blocker therapy reduces long-term mortality in patients with acute ischemic stroke with elevated heart rates. *J. Am. Heart Assoc.* 14:e039678. 10.1161/JAHA.124.039678 40079312 PMC12132726

[B48] Li H. Guo W. Nie Q. Wang Z. Li H. (2026). Central autonomic network dysfunction in Stroke-heart syndrome: Mechanistic roles of the insula and limbic system. *Front. Neurosci.* 20:1690302. 10.3389/fnins.2026.1690302 41788541 PMC12956677

[B49] Li P. Gao Y. Zhao X. (2026). The bone-brain axis: Novel insights into the bidirectional crosstalk in depression and osteoporosis. *Biomolecules* 16:213. 10.3390/biom16020213 41750283 PMC12938012

[B50] Li P. Tao Z. Zhao X. (2024). The role of osteopontin (OPN) in regulating microglia phagocytosis in nervous system diseases. *J. Integr. Neurosci.* 23:169. 10.31083/j.jin2309169 39344228

[B51] Li Z. He X. Fang Q. Yin X. (2024). Gut microbe-generated metabolite trimethylamine-N-oxide and ischemic stroke. *Biomolecules* 14:1463. 10.3390/biom14111463 39595639 PMC11591650

[B52] Lin H. Wei G. Li F. Guo W. Hong P. Weng Y. et al. (2020). Macrophage-NLRP3 inflammasome activation exacerbates cardiac dysfunction after ischemic stroke in a mouse model of diabetes. *Neurosci. Bull.* 36 1035–1045. 10.1007/s12264-020-00544-0 32683554 PMC7475163

[B53] Markousis-Mavrogenis G. Noutsias M. Rigopoulos A. Giannakopoulou A. Gatzonis S. Pons R. et al. (2022). The emerging role of combined brain/heart magnetic resonance imaging for the evaluation of brain/heart interaction in heart failure. *J. Clin. Med.* 11:4009. 10.3390/jcm11144009 35887772 PMC9322381

[B54] McNamara K. Merkler A. Freeman J. Krumholz H. Ahmad T. Sharma R. (2024). Ischemic *Stroke* and reduced left ventricular ejection fraction: A multidisciplinary approach to optimize brain and cardiac health. *Stroke* 55 1720–1727. 10.1161/STROKEAHA.123.045623 38660813

[B55] Meng L. Hou W. Chui J. Han R. Gelb A. (2015). Cardiac output and cerebral blood flow: The integrated regulation of brain perfusion in adult humans. *Anesthesiology* 123 1198–1208. 10.1097/ALN.0000000000000872 26402848

[B56] Mikulenka P. Mihalovic M. Peisker T. Tousek P. (2024). Stroke-heart syndrome - cardiac complications in ischemic stroke patients. *Ceska a Slovenska Neurologie a Neurochirurgie* 87 101–106. 10.48095/cccsnn2024101

[B57] Moura M. Monteiro A. Salgado A. Silva N. Monteiro S. (2024). Disrupted autonomic pathways in spinal cord injury: Implications for the immune regulation. *Neurobiol. Dis.* 195:106500. 10.1016/j.nbd.2024.106500 38614275

[B58] Omerovic E. Citro R. Bossone E. Redfors B. Backs J. Bruns B. et al. (2022). Pathophysiology of Takotsubo syndrome - a joint scientific statement from the heart failure association Takotsubo syndrome study group and myocardial function working group of the european society of cardiology - Part 1: Overview and the central role for catecholamines and sympathetic nervous system. *Eur. J. Heart Fail* 24 257–273. 10.1002/ejhf.2400 34907620

[B59] Packer M. Anker S. Butler J. Filippatos G. Pocock S. Carson P. et al. (2020). Cardiovascular and renal outcomes with empagliflozin in heart failure. *N. Engl. J. Med.* 383 1413–1424. 10.1056/NEJMoa2022190 32865377

[B60] Park H. Kim B. Yoon C. Yang M. Han M. Bae H. (2016). Left ventricular diastolic dysfunction in ischemic stroke: Functional and vascular outcomes. *J. Stroke* 18 195–202. 10.5853/jos.2015.01697 27283279 PMC4901948

[B61] Peh A. O’Donnell J. Broughton B. Marques F. (2022). Gut microbiota and their metabolites in *Stroke*: A double-edged sword. *Stroke* 53 1788–1801. 10.1161/STROKEAHA.121.036800 35135325

[B62] Peixoto A. (2019). Acute severe hypertension. *N. Engl. J. Med.* 381 1843–1852. 10.1056/NEJMcp1901117 31693807

[B63] Pongratz G. Straub R. (2014). The sympathetic nervous response in inflammation. *Arthritis. Res. Ther.* 16:504. 10.1186/s13075-014-0504-2 25789375 PMC4396833

[B64] Redfors B. Jha S. Thorleifsson S. Jernberg T. Angerås O. Frobert O. et al. (2021). Short- and long-term clinical outcomes for patients with Takotsubo syndrome and patients with myocardial infarction: A report from the swedish coronary angiography and angioplasty registry. *J. Am. Heart Assoc.* 10:e017290. 10.1161/JAHA.119.017290 34465127 PMC8649294

[B65] Roberts A. Gu X. Buffa J. Hurd A. Wang Z. Zhu W. et al. (2018). Development of a gut microbe-targeted nonlethal therapeutic to inhibit thrombosis potential. *Nat. Med.* 24 1407–1417. 10.1038/s41591-018-0128-1 30082863 PMC6129214

[B66] Scheitz J. Sposato L. Schulz-Menger J. Nolte C. Backs J. Endres M. (2022). Stroke-heart syndrome: Recent advances and challenges. *J. Am. Heart Assoc.* 11:e026528. 10.1161/JAHA.122.026528 36056731 PMC9496419

[B67] Shinge S. Zhang D. Din A. Yu F. Nie Y. (2022). Emerging Piezo1 signaling in inflammation and atherosclerosis; A potential therapeutic target. *Int. J. Biol. Sci.* 18 923–941. 10.7150/ijbs.63819 35173527 PMC8771847

[B68] Shui X. Chen J. Fu Z. Zhu H. Tao H. Li Z. (2024). Microglia in ischemic stroke: Pathogenesis insights and therapeutic challenges. *J. Inflamm. Res.* 17 3335–3352. 10.2147/JIR.S461795 38800598 PMC11128258

[B69] Simats A. Liesz A. (2022). Systemic inflammation after stroke: Implications for post-stroke comorbidities. *EMBO Mol. Med.* 14 e16269. 10.15252/emmm.202216269 35971650 PMC9449596

[B70] Simats A. Sager H. Liesz A. (2024a). Heart-brain axis in health and disease: Role of innate and adaptive immunity. *Cardiovasc. Res.* 120 2325–2335. 10.1093/cvr/cvae185 39180327

[B71] Simats A. Zhang S. Messerer D. Chong F. Beşkardeş S. Chivukula A. et al. (2024b). Innate immune memory after brain injury drives inflammatory cardiac dysfunction. *Cell* 187 4637–4655.e26. 10.1016/j.cell.2024.06.028 39043180

[B72] Singh T. Khan H. Gamble D. Scally C. Newby D. Dawson D. (2022). Takotsubo syndrome: Pathophysiology, emerging concepts, and clinical implications. *Circulation* 145 1002–1019. 10.1161/CIRCULATIONAHA.121.055854 35344411 PMC7612566

[B73] Stanne T. Angerfors A. Andersson B. Brännmark C. Holmegaard L. Jern C. (2022). Longitudinal study reveals long-term proinflammatory proteomic signature after ischemic *stroke* across subtypes. *Stroke* 53 2847–2858. 10.1161/STROKEAHA.121.038349 35686557 PMC9389938

[B74] Stengl H. Poller W. Di Vece D. Templin C. Endres M. Nolte C. et al. (2024). How the brain impacts the heart: Lessons from ischaemic stroke and other neurological disorders. *Heart* 111 99–108. 10.1136/heartjnl-2024-324173 39515993

[B75] Suberville S. Bellocq A. Fouqueray B. Philippe C. Lantz O. Perez J. et al. (1996). Regulation of interleukin-10 production by beta-adrenergic agonists. *Eur. J. Immunol.* 26 2601–2605. 10.1002/eji.1830261110 8921945

[B76] Sun Y. Zhang X. Jiang Y. Guan X. Li Z. Chu X. et al. (2025). The role of Piezo1 in cardiovascular diseases: From molecular mechanisms to targeted therapeutic potential. *Int. J. Biol. Macromol.* 318:144843. 10.1016/j.ijbiomac.2025.144843 40456339

[B77] Taberner F. Prato V. Schaefer I. Schrenk-Siemens K. Heppenstall P. Lechner S. (2019). Structure-guided examination of the mechanogating mechanism of PIEZO2. *Proc. Natl. Acad. Sci. U S A.* 116 14260–14269. 10.1073/pnas.1905985116 31235572 PMC6628815

[B78] Templin C. Ghadri J. Diekmann J. Napp L. Bataiosu D. Jaguszewski M. et al. (2015). Clinical features and outcomes of takotsubo (Stress) cardiomyopathy. *N. Engl. J. Med.* 373 929–938. 10.1056/NEJMoa1406761 26332547

[B79] Tsai W. Lin H. Lai Y. Hsu C. Huang C. Wang H. et al. (2019). The effect of stroke subtypes on baroreceptor sensitivity, a predict for acute stroke outcome. *Biomed. Res. Int.* 2019:7614828. 10.1155/2019/7614828 31139650 PMC6500628

[B80] Urra X. Cervera A. Obach V. Climent N. Planas A. Chamorro A. (2009). Monocytes are major players in the prognosis and risk of infection after acute stroke. *Stroke* 40 1262–1268. 10.1161/STROKEAHA.108.532085 19164783

[B81] Valenza G. Matić Z. Catrambone V. (2025). The brain-heart axis: Integrative cooperation of neural, mechanical and biochemical pathways. *Nat. Rev. Cardiol.* 22 537–550. 10.1038/s41569-025-01140-3 40033035

[B82] van Nieuwkerk A. Delewi R. Wolters F. Muller M. Daemen M. Biessels G. (2023). Cognitive impairment in patients with cardiac disease: Implications for clinical practice. *Stroke* 54 2181–2191. 10.1161/STROKEAHA.123.040499 37272393

[B83] Wang L. Ma L. Ren C. Zhao W. Ji X. Liu Z. et al. (2024). Stroke-heart syndrome: Current progress and future outlook. *J. Neurol.* 271 4813–4825. 10.1007/s00415-024-12480-4 38869825 PMC11319391

[B84] Wang W. Shih Y. Wei S. Chiu J. (2025). Impacts of aging and fluid shear stress on vascular endothelial metabolism and atherosclerosis development. *J. Biomed. Sci.* 32:83. 10.1186/s12929-025-01177-z 40890841 PMC12403445

[B85] Wen S. Wong C. (2017). An unexplored brain-gut microbiota axis in stroke. *Gut Microbes* 8 601–606. 10.1080/19490976.2017.1344809 28640714 PMC5730388

[B86] Winder K. Villegas Millar C. Siedler G. Knott M. Dörfler A. Engel A. et al. (2023). Acute right insular ischaemic lesions and poststroke left ventricular dysfunction. *Stroke Vasc. Neurol.* 8 301–306. 10.1136/svn-2022-001724 36653066 PMC10512080

[B87] Xu J. Cheng A. Song B. Zhao M. Xue J. Wang A. et al. (2022). Trimethylamine N-oxide and *stroke* recurrence depends on ischemic stroke subtypes. *Stroke* 53 1207–1215. 10.1161/STROKEAHA.120.031443 34794334

[B88] Xu T. Dong F. Zhang M. Wang K. Xu T. Xia S. et al. (2024). Post-stroke arrhythmia could be a potential predictor for post-stroke depression. *Sci. Rep.* 14:9093. 10.1038/s41598-024-59789-8 38643303 PMC11032346

[B89] Zeng W. Marshall K. Min S. Daou I. Chapleau M. Abboud F. et al. (2018). PIEZOs mediate neuronal sensing of blood pressure and the baroreceptor reflex. *Science* 362 464–467. 10.1126/science.aau6324 30361375 PMC6563913

[B90] Zha Z. Cheng Y. Cao L. Qian Y. Liu X. Guo Y. et al. (2021). Monomeric CRP aggravates myocardial injury after myocardial infarction by polarizing the macrophage to pro-inflammatory phenotype through JNK signaling pathway. *J. Inflamm Res.* 14 7053–7064. 10.2147/JIR.S316816 34984018 PMC8703048

[B91] Zhang Y. Huang K. Duan J. Zhao R. Yang L. (2024). Gut microbiota connects the brain and the heart: Potential mechanisms and clinical implications. *Psychopharmacology.* 241 637–651. 10.1007/s00213-024-06552-6 38407637

[B92] Zhou L. Wang T. Yu Y. Li M. Sun X. Song W. et al. (2022). The etiology of poststroke-depression: A hypothesis involving HPA axis. *Biomed. Pharmacother*. 151:113146. 10.1016/j.biopha.2022.113146 35643064

[B93] Zhu L. Huang L. Le A. Wang T. Zhang J. Chen X. et al. (2022). Interactions between the autonomic nervous system and the immune system after stroke. *Compr. Physiol.* 12 3665–3704. 10.1002/cphy.c210047 35766834

[B94] Zhu W. Gregory J. Org E. Buffa J. Gupta N. Wang Z. et al. (2016). Gut microbial metabolite TMAO enhances platelet hyperreactivity and thrombosis risk. *Cell* 165 111–124. 10.1016/j.cell.2016.02.011 26972052 PMC4862743

[B95] Ziaka M. Exadaktylos A. (2023). The heart is at risk: Understanding stroke-heart-brain interactions with focus on neurogenic stress cardiomyopathy-a review. *J. Stroke* 25 39–54. 10.5853/jos.2022.02173 36592971 PMC9911836

[B96] Zou P. Li T. Cao Z. Yang E. Bao M. Zhang H. et al. (2025). High-altitude hypoxia aggravated neurological deficits in mice induced by traumatic brain injury via BACH1 mediating astrocytic ferroptosis. *Cell. Death Discov.* 11:46. 10.1038/s41420-025-02337-8 39905004 PMC11794473

